# Analysis of Urine

**Published:** 1883-07

**Authors:** 


### ANALYSIS OF URINE.

Since opening our laboratory here, for the systematic examination of urine, the unexpectedly large amount sent us, even from distant points, is a most gratifying indication of the already large appreciation by the profession of this means of diagnosis.

The truth is—all consideration of self out of view—the time is past when a physician, who has any respect for his intelligence, much more conscience, can afford to be indifferent to this invaluable source of diagnostic information.

We commend to the readers of the REGISTER the following quotation from a recent lecture of Dr. Pepper, (*Phila. Med. Times*): "As I have so frequently said to you in connection with the examination of patients, I am sure that you will never arrive at any degree of accuracy in your diagnosis unless you adopt the infallible rule of examining the urine of every individual who consults you, no matter what may be the symptoms present."

